# StarD7 Knockdown Modulates ABCG2 Expression, Cell Migration, Proliferation, and Differentiation of Human Choriocarcinoma JEG-3 Cells

**DOI:** 10.1371/journal.pone.0044152

**Published:** 2012-08-29

**Authors:** Jésica Flores-Martín, Viviana Rena, Sebastián Márquez, Graciela M. Panzetta-Dutari, Susana Genti-Raimondi

**Affiliations:** Centro de Investigaciones en Bioquímica Clínica e Inmunología-Consejo Nacional de Investigaciones Científicas y Técnicas, Departamento de Bioquímica Clínica, Facultad de Ciencias Químicas, Universidad Nacional de Córdoba, Córdoba, Argentina; Virgen Macarena University Hospital, Spain

## Abstract

**Background:**

StAR-related lipid transfer domain containing 7 (StarD7) is a member of the START-domain protein family whose function still remains unclear. Our data from an explorative microarray assay performed with mRNAs from StarD7 siRNA-transfected JEG-3 cells indicated that ABCG2 (ATP-binding cassette sub-family G member 2) was one of the most abundantly downregulated mRNAs.

**Methodology/Principal Findings:**

Here, we have confirmed that knocking down StarD7 mRNA lead to a decrease in the xenobiotic/lipid transporter ABCG2 at both the mRNA and protein levels (−26.4% and −41%, *p*<0.05, at 48 h of culture, respectively). Also a concomitant reduction in phospholipid synthesis, bromodeoxyuridine (BrdU) uptake and ^3^H-thymidine incorporation was detected. Wound healing and transwell assays revealed that JEG-3 cell migration was significantly diminished (*p*<0.05). Conversely, biochemical differentiation markers such as human chorionic gonadotrophin β-subunit (βhCG) protein synthesis and secretion as well as βhCG and syncytin-1 mRNAs were increased approximately 2-fold. In addition, desmoplakin immunostaining suggested that there was a reduction of intercellular desmosomes between adjacent JEG-3 cells after knocking down StarD7.

**Conclusions/Significance:**

Altogether these findings provide evidence for a role of StarD7 in cell physiology indicating that StarD7 modulates ABCG2 multidrug transporter level, cell migration, proliferation, and biochemical and morphological differentiation marker expression in a human trophoblast cell model.

## Introduction

StarD7 belongs to the family of START proteins ubiquitously expressed, which are implicated in lipid transport, metabolism, and signaling [1]–[3]. StarD7 mRNA was first identified using differential display techniques as a JEG-3 over-expressed gene compared with normal and benign trophoblastic samples [4]. In subsequent experiments, we demonstrated a predominant cytoplasmic localization of StarD7 in human cytotrophoblast cells with a clear and partial re-localization towards the plasma membrane after the syncytialization process [5]. Moreover, recombinant StarD7 accelerates bilayer fusion between donor and acceptor liposomes, induces multinuclear giant BeWo cell formation [6], and is able to form stable Gibbs and Langmuir monolayers at the air-buffer interface, showing a marked interaction with phospholipid monolayers [7]. A recent study reported that StarD7 protein facilitates the delivery of phosphatidylcholine to the mitochondria [8]. Also, we demonstrated that the regulation of StarD7 gene expression in JEG-3 cells occurs through a steroidogenic transcription factor 1 (SF-1) and β-catenin-mediated activation mechanism that synergistically activate StarD7 promoter [9], [10]. Subsequently, in order to get insight into the function of StarD7 in cell physiology, exploratory differential gene expression analysis of JEG-3 cells transfected with StarD7 siRNA was performed by microarray experiments. Data analysis indicated that ABCG2 was one of the most abundantly downregulated mRNAs [11].

ABCG2 is a member of the ABC protein superfamily of multidrug efflux transporters [12]. ABCG2 is an integral plasma membrane glycoprotein distributed in normal human tissues and particularly highly expressed in those with barrier function, including the placenta, testes, liver, kidney, intestine and brain [13], [14]. ABCG2 is overexpressed in tumors, cancer cell lines and in a subpopulation of stem cells: the side populations, conferring multidrug resistance [15], [16]. Alterations in ABCG2 expression linked with changes in cell proliferation, migration and invasion have been reported [17]–[19]. Besides the role as a drug and xenobiotic transporter or fetus protection against potential toxicity [20], [21], other physiological functions of ABCG2 in the placenta have been proposed [13], [14]. A role of ABCG2 in the transverse distribution of lipids in the plasma membrane during trophoblast syncytialization has been suggested [22]. *In vitro* trophoblast fusion and differentiation were accompanied with significantly increased in ABCG2 expression [23]; while inhibition of ABCG2 activity caused cytokine-induced trophoblast cell apoptosis [24]. Therefore, it has been suggested that the lower placental ABCG2 expression found in intrauterine growth retardation pregnancies may cause a deficit in placental function and survival [24].

In light of the above information, the present study was undertaken to establish the impact of StarD7 siRNA on ABCG2 expression in JEG-3 cells in connection with cell migration and proliferation. Moreover, phospholipids synthesis and biochemical and morphological JEG-3 cell differentiation markers were analyzed.

## Results

### StarD7 siRNA Decreases ABCG2 mRNA and Protein Levels

**Figure 1:**
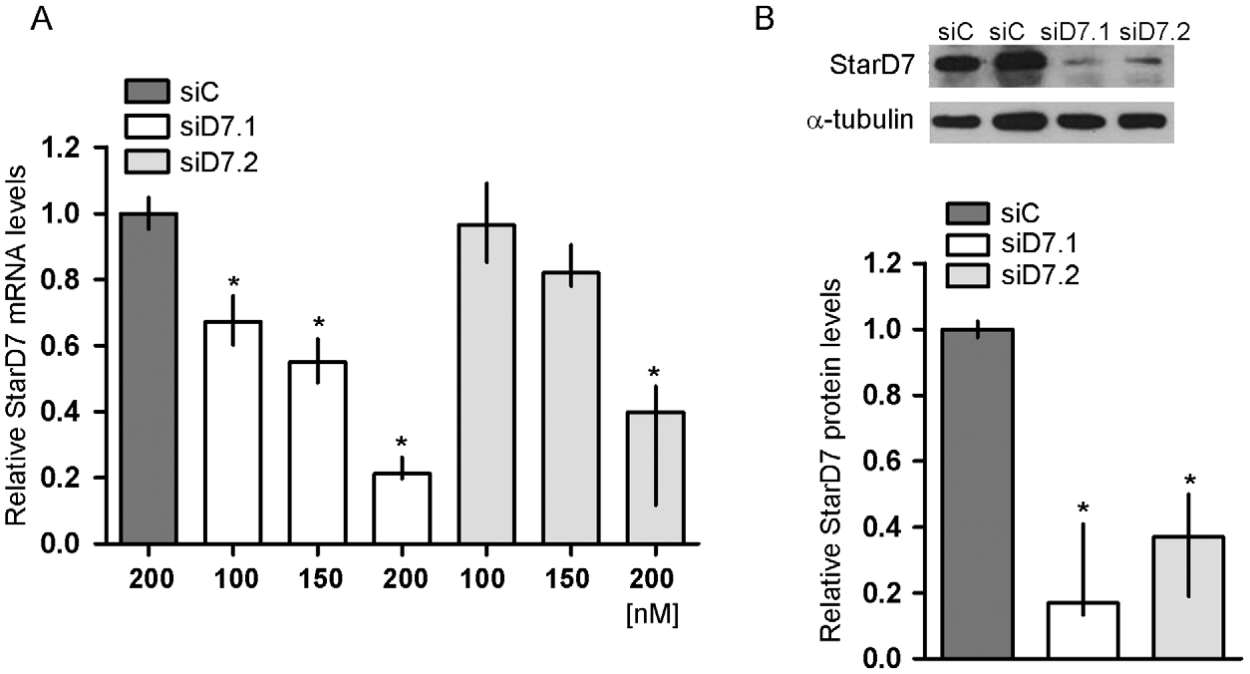
Effect of StarD7 siRNA on StarD7 mRNA and protein expression in JEG-3 cells. Cells were transfected with 100, 150 and 200 nM of StarD7.1 (siD7.1) or StarD7.2 (siD7.2) siRNAs and cultured for 48 h. Control cells were transfected with 200 nM of scrambled siRNA (siC). **A**-The StarD7 expression was determined by real-time quantitative PCR. Results are expressed as StarD7 mRNA expression in StarD7 siRNA-transfected cells after normalizing to cyclophilin A relative to the corresponding normalized mRNA levels in scrambled siRNA-transfected cells. The values represent the median and 25^ th^–75^ th^% percentiles of at least three independent experiments performed by triplicate. **B**- StarD7 protein expression was analyzed by western blot. Protein extracts (100 µg/lane) from scrambled siRNA- (siC), StarD7.1 siRNA- (siD7.1) or StarD7.2 siRNA- (siD7.2) transfected cells were electrophoresed on a 7.5% SDS polyacrylamide gel and transferred to a nitrocellulose filter. Filters were incubated with anti-StarD7Ct antibody (top) and with the monoclonal anti-α-tubulin antibody (bottom). A representative blot of at least five independent experiments with similar results is shown. The bar graph represents the densitometric quantification of StarD7 protein levels in StarD7 siRNA-transfected cells normalized to α-tubulin of five separate experiments relative to the corresponding normalized protein levels in scrambled siRNA-transfected cells defined as 1 (median and 25^ th^–75^ th^% percentiles). *p<0.05 compared to scrambled siRNA-transfected cells.

To elucidate the impact of StarD7 siRNA on ABCG2 expression, JEG-3 cells were transfected with two different sets of double-stranded siRNA designed against different sequences of the StarD7 mRNA (Table 1). Among the two StarD7 siRNAs, StarD7.1 siRNA appeared a little more effective to inhibit StarD7 mRNA expression in JEG-3 cells, but no statistically significant difference among them was observed at all concentrations analyzed (p>0.05). After 48 h post transfection, approximately up to −79% and −60% (*p*<0.05) reduction of StarD7 mRNA expression was observed in JEG-3 cells treated with 200 nM of StarD7.1 and StarD7.2 siRNAs, respectively (Fig. 1A). In addition, approximately up to −83% and −63% (*p*<0.05) inhibition of StarD7 expression was detected by western blot with 200 nM of StarD7.1 siRNA or StarD7.2 siRNA, respectively (Fig. 1B). In the following experiments only the results obtained with StarD7.1 siRNA are shown, even though similar results were observed with StarD7.2 siRNA.

**Table 1:** Sequence of double-stranded StarD7 siRNA.

	Sequence	Position
**StarD7.1 siRNA**		
Sense	GGUAUAGUGUGGAUCAGGATT	nucleotides 1099–1117
Antisense	UCCUGAUCCACACUAUACCGC	
**StarD7.2 siRNA**		
Sense	GCACCCACCUUUACCAGUATT	nucleotides 877–895
Antisense	UACUGGUAAAGGUGGGUGCCT	

**Figure 2:**
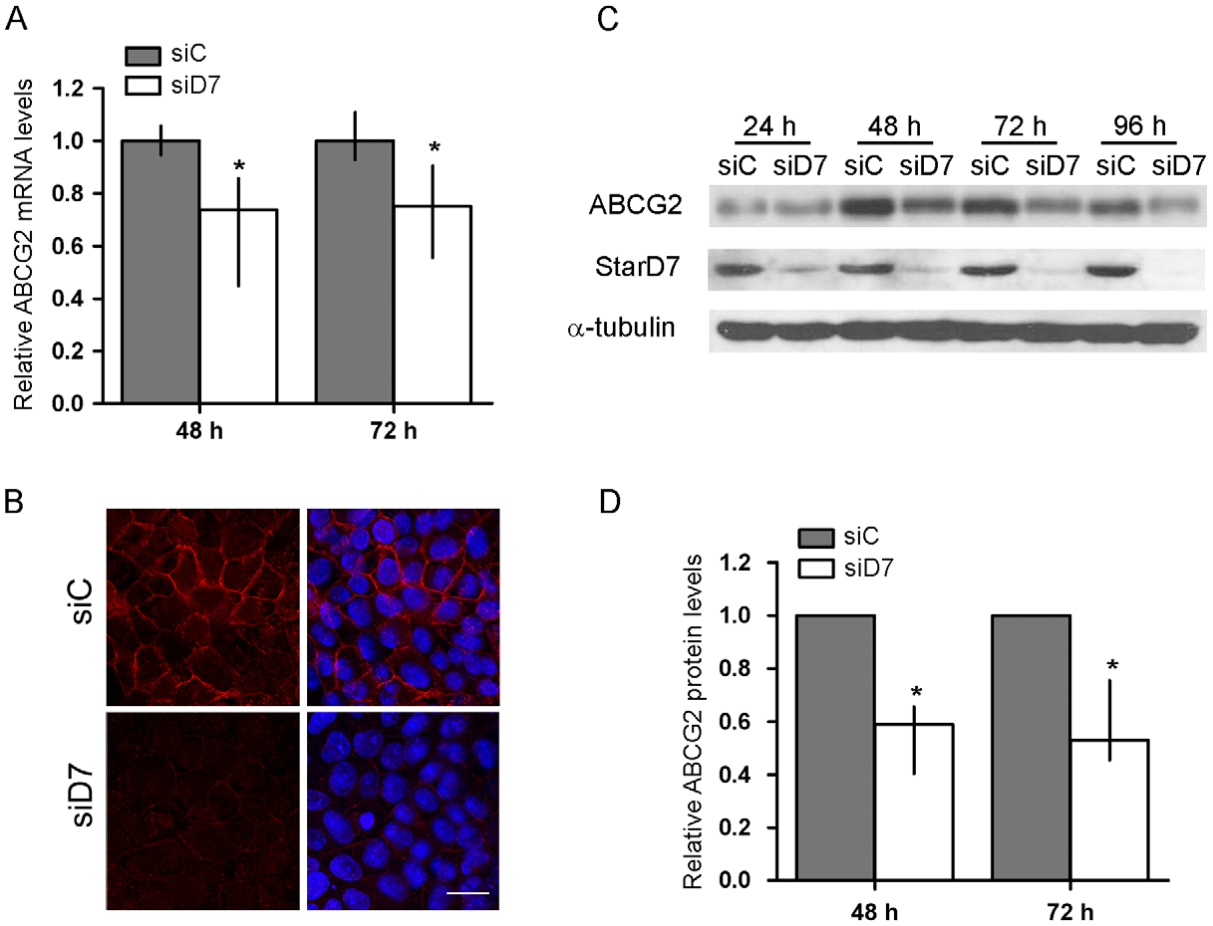
Effect of StarD7 siRNA on ABCG2 mRNA and protein expression in JEG-3 cells. **A**- Cells were transfected with 200 nM of StarD7.1 or scrambled siRNAs and cultured for 48 h or 72 h. ABCG2 expression was determined by real-time quantitative PCR. Results are expressed as ABCG2 mRNA expression in StarD7 siRNA-transfected cells after normalizing to cyclophilin A relative to the corresponding normalized mRNA levels in scrambled siRNA-transfected cells. The values represent the median and 25^ th^–75^th^% percentiles of at least three independent experiments performed by triplicate. B- Confocal microscopy images demonstrating membrane expression of ABCG2 protein (red) in scrambled siRNA-treated JEG-3 cells (siC, upper panel) and low or absent ABCG2 immunostaining in StarD7 siRNA-treated cells (siD7, bottom panel). The nuclei were labelled with Hoescht (blue) and merge images are shown on the right. Bar  = 20 µm (×600). **C**- Cells were transfected with 200 nM of StarD7.1 siRNA or 200 nM of scrambled siRNA and cultured until 96 h. ABCG2 protein expression was analyzed by western blot. Protein extracts (100 µg/lane) from scrambled siRNA- (siC) or StarD7 siRNA-transfected (siD7) cells were electrophoresed on a 7.5% SDS polyacrylamide gel and transferred to a nitrocellulose filter. Filters were incubated with anti-ABCG2 (top), anti-StarD7Ct (middle) and with the monoclonal anti-α-tubulin antibodies (bottom). These immunoblots are representative of at least three separate experiments. **D**- The bar graph represents the densitometric quantification of ABCG2 protein levels in StarD7 siRNA-transfected cells normalized to α-tubulin of at least four separate experiments relative to the corresponding normalized protein levels in scrambled siRNA-transfected cells (median and 25^th^–75^th^% percentiles). *p<0.05 compared to scrambled siRNA-transfected cells.

StarD7 knockdown led to a significant reduction in ABCG2 mRNA and protein levels in JEG-3 cells transfected with StarD7 siRNA as compared to scrambled siRNA-transfected ones. A significant inhibition of ABCG2 mRNA expression was observed in cells transfected with 200 nM of StarD7 siRNA cultured for 48 h or 72 h (Fig. 2A, *p*<0.05). In addition, western blot assays performed with protein extracts from StarD7 siRNA-treated cells revealed approximately up to −41% and −47% (*p*<0.05) diminution of ABCG2 protein level from 48 h of transfection, in parallel with a reduction in StarD7 protein level (Fig. 2C and 2D). Furthermore, immunofluorescence assays indicated that ABCG2 was localized to the plasma membrane of JEG-3 cells treated with scrambled siRNA but low or almost absent immunofluorescent ABCG2 signal was detected in the StarD7 siRNA-treated cells (Fig. 2B). Collectively, these data demonstrate that StarD7 siRNA results in a significant diminution of ABCG2 expression at both transcript and protein levels.

### StarD7 siRNA Diminishes JEG-3 Cell Injury Repair, Migration, and Proliferation

**Figure 3:**
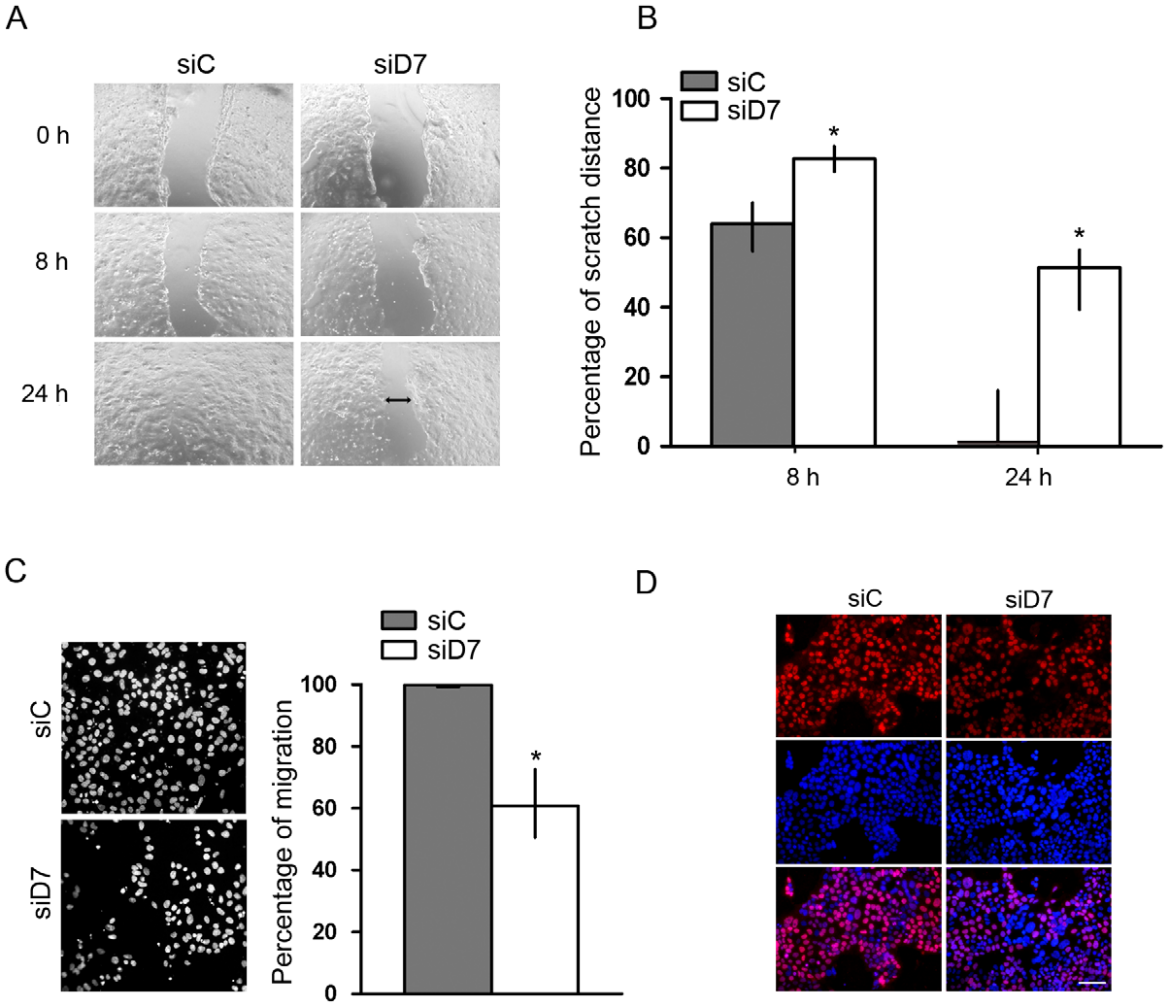
Effect of StarD7 silencing on JEG-3 cell migration. **A**- Wound healing assay in JEG-3 cells treated with StarD7.1 (siD7) or scrambled (siC) siRNAs. An open furrow was generated by scratching confluent cells using a pipette tip. Confluency was restored in controls after 24 h. However, in cells treated with StarD7 siRNA, confluency was not restored after 24 h. **B**- The distance between furrow edges in the scrambled or StarD7 siRNA-treated cells of three independent experiments was measured and presented graphically as percentage of the initial distance (0 h);*p<0.05 compared to scrambled siRNA-transfected cells. **C**- Transwell *In vitro* migration assays. Left panels: representative images show cells migrated to the lower chamber after 48 hours (×200). Right panels: Bar graph represents the percentage of cell migration in seven fields of duplicate wells containing StarD7 siRNA-treated cells relative to scrambled siRNA ones (median and 25^th^–75^th^% percentiles, n = 3); *p<0.05 compared to scrambled siRNA-transfected cells. **D-**Cell proliferation was determined by BrdU (red) staining of JEG-3 cells treated with scrambled (left panel) or StarD7.1 (right panel) siRNAs. The nuclei were labelled with Hoescht (blue, middle panels) and merge images are shown on the bottom panels. Bar  = 50 µm (×200). The images are representative of three experiments with consistent results.

Since it has been reported that alterations in ABCG2 expression are related with changes in cell migration and proliferation [17]–[19], we explored the effect of StarD7 reduction on cell migration performing monolayer wounding assay. Similar sized wounds were introduced in JEG-3 cell monolayers at 0 h. As shown in Figure 3A, in scrambled siRNA-treated cells the gap of the wound was gradually filled by migrating cells, and 24 h after wound the gap was almost closed. In contrast, transfection of StarD7 siRNA induced a decrease in cell migration. The speed of wound closure was much slower and the gap remained widely open after 24 h of culture. Figure 3B shows a quantitative analysis of pooled data, confirming that the remarkable difference in JEG-3 cell wound healing as a consequence of siRNA-mediated StarD7 knockdown was significant (*p*<0.05, n = 3). In addition, transwell assays with StarD7 siRNA-transfected cells showed a significant decrease in JEG-3 cell migration up to 40% of that observed in cells treated with scrambled siRNA (Fig. 3C). The quantitative analyses of pooled data document a clear inhibition of cell migration following StarD7 reduction.

To test whether this change in cell migration was accompanied with alterations in cell proliferation the BrdU uptake assay was performed. As shown in Figure 3D, there was a lower number of BrdU-positive cells in cultures treated with StarD7 siRNA than in scrambled siRNA-treated ones 40.7% (range 36.1–49.6%) vs 68% (range 64.1–74.0%), respectively (median and 25^th^–75^th^% percentiles, *p*<0.05, n = 3). Importantly, StarD7 reduction results in an inhibition of JEG-3 cell proliferation as indicated by the ^3^H-thymidine incorporation into StarD7 siRNA-treated cells compared to scrambled siRNA-treated ones. A significant reduction of −27% (25^th^–75^th^% percentiles: 18–41%, *p*<0.05) was obtained from three experiments performed in quintuplicate.

### StarD7 Reduction is Associated with Decreased Phospholipid Synthesis

**Figure 4:**
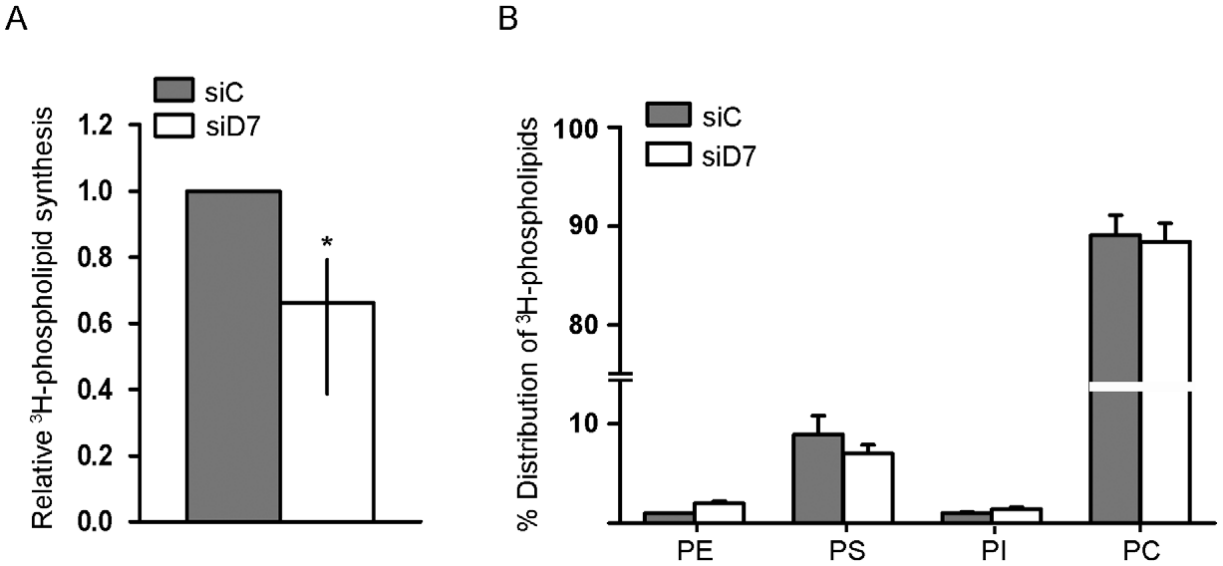
The *de novo* biosynthesis of total glycerophospholipids in StarD7 siRNA-treated JEG-3 cells. **A**- Relative ^3^H-glycerophospholipid synthesis in StarD7.1 (siD7) siRNA-treated JEG-3 cells compared to scrambled (siC) siRNA-treated cells defined as 1. Data are median and 25^th^–75^th^% percentiles (*n* = 4 independent experiments). *p<0.05 compared to scrambled siRNA-transfected cells. **B**- Percentage of distribution of major individual ^3^H-phospholipids in StarD7 siRNA-treated JEG-3 cells or in scrambled siRNA cells. Data are mean ± SEM (*n* = 2 independent experiments). Phosphatidylcholine (PC) is the species with the highest precursor incorporation in both cell conditions, followed by phosphatidylserine (PS) and after phosphatidylinositol (PI) and phosphatidylethanolamine (PE) with a minor percentage.

Mounting evidence indicates a strong connection among ABC transporter function and phospholipid synthesis, metabolism and signaling [25], [26]. Moreover, it has been reported that StarD2 promotes ATP-binding cassette protein A1-mediated efflux of cholesterol and phosphatidylcholine molecules as nascent pre-β-high-density lipoprotein particles [27]. Thus, to further determine the functional significance of the StarD7 reduction in JEG-3 cells we examined whether the de novo phospholipid synthesis is altered by StarD7 siRNA. For this aim, we examined ^3^H-glycerol incorporation into phospholipids in StarD7 siRNA- and scrambled-transfected cells after 48 hours of culture (Fig. 4A and B). Results show that there was a significant reduction in the biosynthesis of total phospholipids in JEG-3 cells treated with StarD7 siRNA compared to those treated with scrambled siRNA (Fig. 4A). However, the percentage of distribution of the major phospholipid species: phosphatidylcholine, phosphatidylserine, phosphatidylinositol, and phosphatidylethanolamine remained unchanged; being phosphatidylcholine the most abundant phospholipid synthesized in both experimental conditions (Fig. 4B). These results suggest that StarD7 downregulation leads to a global reduction in the synthesis of the main phospholipid species.

### StarD7 siRNA Increases the Expression of Biochemical Differentiation Markers

**Figure 5:**
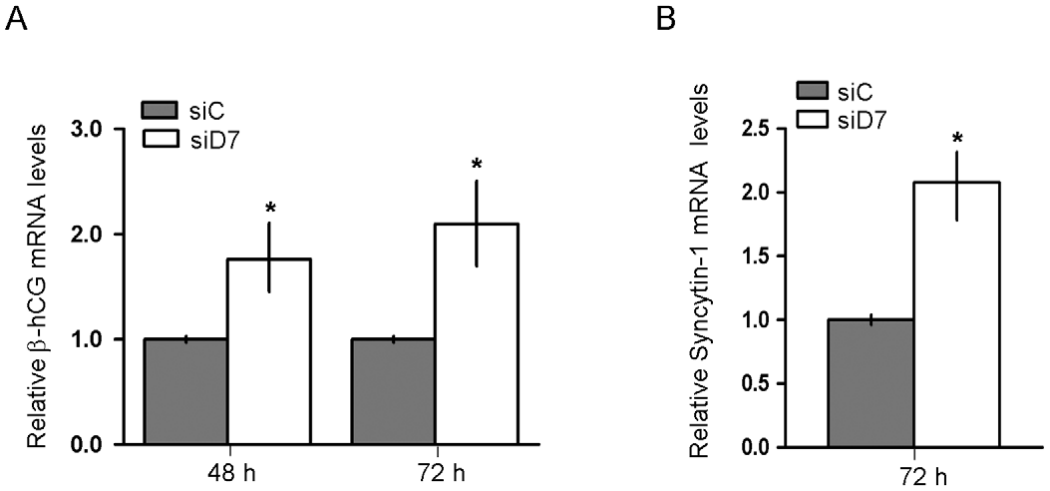
βhCG and syncytin-1 mRNA expression are upregulated in JEG-3 cells by StarD7 silencing. Quantitative RT-PCR analysis of βhCG (**A**) and syncytin-1 (**B**) mRNAs in siRNAs-treated JEG-3 cells is shown. Analysis was performed using cDNAs derived from one µg of total RNA extracted from cells treated with StarD7.1 (siD7) or scrambled (siC) siRNA and cultured until 72 hours. Results are expressed as mRNA expression in StarD7siRNA-transfected cells after normalizing to cyclophilin A relative to the corresponding normalized mRNA levels in scrambled siRNA-transfected cells. The values represent the median and 25^th^–75^th^% percentiles of triplicate results obtained from at least three independent experiments; *p<0.05 compared to scrambled siRNA-transfected cells.

**Figure 6:**
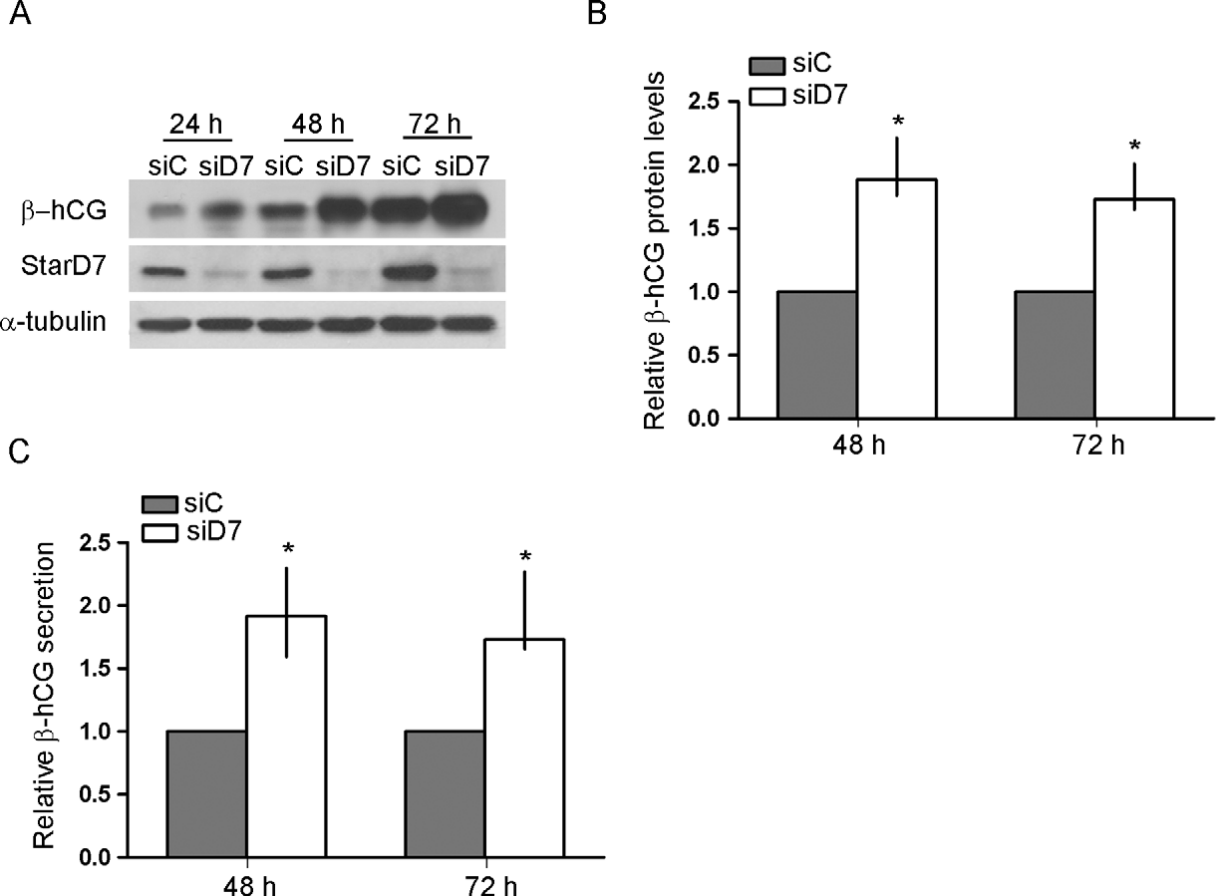
βhCG protein expression is upregulated in JEG-3 cells by StarD7 silencing. Cells were transfected with scrambled or StarD7.1 siRNAs for 6 h and then cultured until 72 hours. **A**- βhCG and StarD7 protein expression were analyzed by western blot. Protein extracts (100 µg/lane) from StarD7.1 siRNA-transfected (siD7) or scrambled siRNA-transfected (siC) cells were electrophoresed on a 7.5% SDS polyacrylamide gel and transferred to a nitrocellulose filter. Filters were incubated with anti-βhCG (top), anti-StarD7Ct (middle) and with the monoclonal anti-α-tubulin antibodies (bottom). These immunoblots are representative of at least three separate experiments. **B**- The bar graph represents the densitometric quantification of βhCG protein levels in StarD7 siRNA-transfected JEG-3 cells normalized to α-tubulin of five separate experiments relative to the corresponding normalized protein levels in scrambled siRNA-transfected cells defined as 1 (median and 25^th^–75^th^% percentiles). **C**- hCG secretion from JEG-3 cells after 48 h or 72 h of culture in StarD7.1 siRNA-transfected condition compared to scrambled siRNA-transfected cell culture defined as 1 (n = 5). *p<0.05 compared to scrambled siRNA-transfected cells.

It has been reported that ABCG2 silencing lead to a decrease in the expression of the biochemical differentiation markers syncytin and hCG in BeWo cells, protecting them during the period of transient membrane instability associated to biochemical differentiation and fusion events [22]. Thus, we next explored whether βhCG and syncytin-1 mRNAs levels are modified by knocking down StarD7. Surprisingly, quantitative RT-PCR analysis data showed an enhancement in both βhCG and syncytin-1 transcripts in StarD7 siRNA- compared to scrambled siRNA-transfected JEG-3 cells (Fig. 5, *p*<0.05). Next, we analyzed whether the increase in βhCG mRNA was also reflected in an increase in βhCG protein levels. A clear and significant increase in βhCG synthesis was observed in JEG-3 cells by StarD7 silencing at all time points analyzed (Fig. 6A and 6B, *p*<0.05). An evident negative correlation between StarD7 and βhCG levels was observed (Fig. 6A, middle panel). These results were also concurrent with an increase in βhCG secretion in StarD7 siRNA-transfected cells consistent with a biochemical differentiation of JEG-3 cells, compared to cells treated with scrambled siRNA (Fig. 6C, *p*<0.05).

### StarD7 siRNA Increases Formation of Syncytial-like Structures

**Figure 7:**
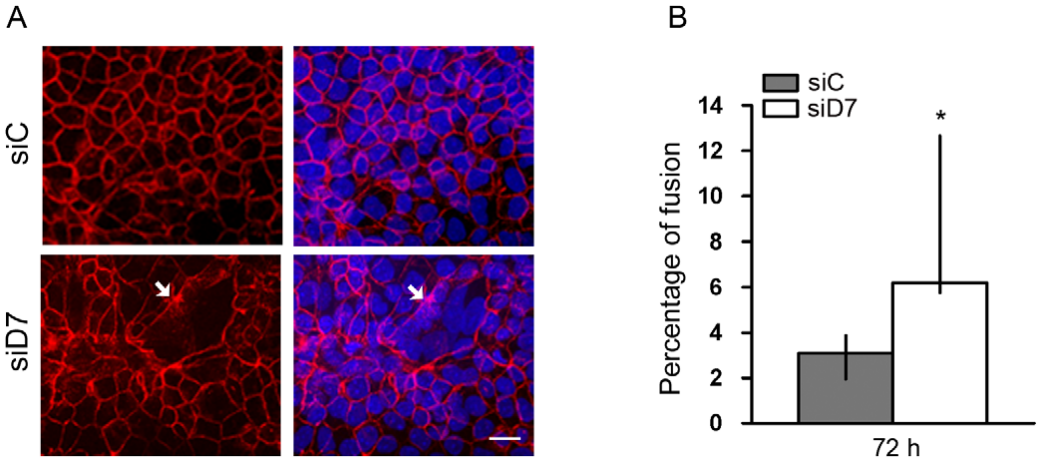
Effect of StarD7 knockdown on JEG-3 cell differentiation. Cells were transfected with scrambled (siC) or StarD7.1 (siD7) siRNAs for 6 h and then cultured until 72 hours. **A**- Detection of desmoplakin (red) in siRNA-treated JEG-3 cells was performed by immunofluorescence (left panel). The nuclei were labelled with Hoescht (blue) and merge images are shown on the right. Syncytial structures were characterized by the absence or incomplete desmoplakin staining (arrows). In control cells, desmoplakin labeling was continuous at the periphery of cells. Bar = 20 µm (×400). **B**- Percentage fusion was determined in cells transfected with StarD7.1 or scrambled siRNAs and represents the percentage of nuclei number in syncytia. Twenty fields were counted for each condition in three different experiments performed in duplicate as described in Materials and Methods. Results are depicted in terms of median percentage and 25^th^–75^th^% percentiles; *p<0.05 compared to scrambled siRNA-transfected cells.

To explore whether the increase in βhCG and syncytin-1 expression was linked to alterations in cell morphology, desmoplakin staining was performed. As shown in Figure 7A StarD7 gene knockdown induced morphological changes in JEG-3 cells, promoting formation of syncytium-like structures. Even though a small cellular fusion index was detected in StarD7 siRNA-transfected cells (8%), it represents an almost three-fold increase as compared to cells transfected with scrambled siRNA (Fig. 7B, *p*<0.05).

## Discussion

StarD7 belongs to the START protein family widely expressed in human cell lines, with highest levels in JEG-3, JAR, HepG2, and HT-29 cell lines [4]. This family of proteins has been implicated in lipid transport, metabolism, and signaling [3], [28]. Human StarD7 orthologous genes have been annotated in the Ensembl and GenBank databases in different genomes, suggesting a conserved physiological function [29]. StarD7 protein has been predicted in most animal phyla, vertebrates and invertebrates as well as in plants highlighting its functional role. Even though some studies have been performed, its physiological function has not been fully elucidated [5], [8]. Here, we investigated the effect of reduced StarD7 expression by siRNA on different aspects of JEG-3 cell physiology. First, we established that StarD7 knockdown led to a decreased expression at both mRNA and protein levels for the ABCG2 xenobiotic/lipid transporter, concurrently with a significant diminution in cell migration, cell proliferation and phospholipid biosynthesis. Furthermore, βhCG mRNA expression as well as its protein synthesis and secretion were increased. This effect was correlated with a concomitant induction of the endogenous syncytin-1 mRNA level and a slight but significant reduction of intercellular desmosomes between adjacent JEG-3 cells. All together, these findings suggest that StarD7 modulates important aspects of trophoblast cell physiology.

The fact that ABCG2 expression was decreased after down-regulation of StarD7 draw our attention due to the important functions described for ABCG2 in cell physiology. The primary biological role of ABCG2 is to protect the organism from insults by a range of toxic, drug or carcinogenic xenobiotics, forming an important defense barrier. Apart from their function in drug transport and cell protection, ABCG2 and some other ABC proteins have been implicated to regulate phospholipid asymmetry by trafficking structural lipids within plasma membranes [25], [26], [30], [31]. Moreover, ABCG2 and ABCC1 participate in the secretion of the bioactive sphingosine 1-phosphate (S1P) [25]. Evseenko et al. demonstrated that the reduction of ABCG2 expression in BeWo cells by siRNA resulted in a manifest increase of phosphatidylserine externalization with ceramide accumulation. These effects were accompanied with a decrease of βhCG and syncytin mRNAs when cells were induced to differentiate by forskolin treatment; results that appear somehow in contradiction with the present data. Even though in both studies there was a down regulation of ABCG2 levels, it is important to emphasize that in the present report ABCG2 diminution was achieved after that StarD7 expression was knocked down. In addition, the observed discrepancies could be understood considering that Evseenko’s study was performed on differentiating BeWo cells while undifferentiating JEG-3 cells were employed in the present study. Furthermore, both cell lines differ in several characteristics such as proliferative activity, degree of differentiation, cell motility, migration, and oxidative stress gene expression [32]–[34].

The specific mechanism by means StarD7 siRNA impact on JEG-3 cell migration and cell proliferation is unknown at present. However, it might be related to the reduction of ABCG2 expression. Indeed, several reports link alterations in ABCG2 expression with changes in cell migration, in addition to invasion and proliferation. Numerous studies documented that overexpression of ABCG2 correlates with higher migration and invasion in a variety of different tumor types [17], [35], [36]. On the contrary, inhibition of ABCG2 resulted in an impaired migration and tube formation of human microvascular endothelial cells [18] and also in inhibition of cellular proliferation in cancer cell lines [16]. Additionally, it was reported that hsa-miR-520 h downregulates ABCG2 in pancreatic cancer cells leading to inhibition of migration, invasion, and side population cells [19].

It has been established that ABCG2 activity is regulated by caveolin-1 through protein-protein interactions and also by membrane cholesterol micro-domain interactions [37], [38], underscoring the relevance of cellular lipid content, metabolism and distribution in ABCG2 function. Phospholipids are predominantly synthesized in the endoplasmic reticulum and subsequently transported to various destinations by vesicular transport through the fusion of vesicles to an acceptor compartment or can also be delivered to specific cellular organelles by monomeric exchange [39], [40]. In this regard, Horibata et al. reported that StarD7 mediates the delivery of phosphatidylcholine to mitochondria [8]. We have previously described that, in *In vitro* differentiating cytotrophoblast cells, StarD7 shows a partial re-localization towards the plasma membrane, suggesting that it could be implicated in the delivery of lipids to the cellular membrane [5]. Although we have not found modifications in the percentage of distribution of the main individual lipid species analyzed we cannot rule out a change in the amount of other minority compounds or alterations in the subcellular localization of particular phospholipids. Thus, it is possible to hypothesize that the observed phospholipid biosynthesis diminution is compatible with a compensatory mechanism aimed at reducing phospholipid accumulation, as a result of a decrease in phospholipid transport between organelles. Phosphatidylcholine, the main intracellular phospholipid, is metabolized to phosphatidic acid which in turn is converted to lysophosphatidate (LPA). Phosphatidic acid can also lead to sphingosine kinase-1 activation, which biotransforms sphingosine to S1P. LPA and S1P are survival signals that promote proliferation, migration, survival and angiogenesis [25]. In this regard, it has been reported a decline in the intracellular sphingosine concentration and sphingosine kinase 1 expression during trophoblast syncytialization [41], [42]. Moreover, the addition of S1P to cultured cytotropholasts led to a reduction in hCG secretion [42]. Our results indicate that the effect of StarD7 knockdown on total phospholipid biosynthesis diminution was accompanied with a decrease in cell migration and proliferation, and an increase in JEG-3 cell fusion and in the biochemical differentiation marker expression, βhCG and syncytin-1. In this scenario, even though we did not measure the intracellular level of S1P, it is possible to consider that phospholipid biosynthesis diminution led to a decline in S1P concentration which in turn stimulated the syncytialization process and also negatively regulated cell migration and proliferation. This hypothesis and our data are in line with the diminution in radiolabeled glycerol incorporation into the novo triacylglycerol and phospholipid biosynthesis during cytotrophoblast cell culture differentiation [43].

Herein, we observed that StarD7 downregulation in the choriocarcinoma JEG-3 cells induces cell fusion and expression of βhCG and syncytin. In contrast, in the context of normal cytotrophoblast cells that undergo spontaneous in vitro syncytialization, StarD7 mRNA and protein expression was increased [5]. These data suggest that an appropriate StarD7 level is required for normal cell physiology. There are several reports that support this assumption. First, StarD7 was originally identified as an up-regulated gene in the choriocarcinoma JEG-3 cell line with respect to their nonmalignant counterpart, complete hydatidiform mole and normal trophoblastic tissue [4]. Overexpressed StarD7 gene was one of the 147 genes specifically associated with colorectal tumor cells [44]. In addition, StarD7 upregulation has also been reported in B-chronic lymphocytic leukemia [45] and in multiple cancer cell lines [4]. Interestingly, Ikeda et al. found that miR-193b inhibits pancreatic cancer cell proliferation concomitantly with its ability to target and downregulate StarD7 transcript expression in pancreatic cells, effect associated with an inhibition in cellular proliferation [46]. Finally, StarD7 gene promoter is activated by Wnt/β-catenin signaling [10], a pathway that promotes proliferation and is frequently altered in cancer cells. These considerations and the observed induction of βhCG synthesis and secretion by StarD7 silencing are in line with the fact that several pathologic alterations in syncytiotrophoblast function have been associated to high hCG production and secretion [47]–[49]. Therefore, dysregulation of StarD7 expression could result in an altered trophoblast function or differentiation leading to an increased risk of placental disorders.

In summary, this study reveals that the loss of StarD7 protein in JEG-3 cells alters ABCG2 multidrug transporter level, cell migration, cell proliferation, and differentiation marker expression providing evidence for a new role for StarD7 in trophoblast cell physiology.

## Materials and Methods

### Antibodies

Mouse monoclonal anti-human ABCG2 (BXP-21 sc-58222) was obtained from Santa Cruz, polyclonal rabbit anti-human chorionic gonadotropin β-subunit (βhCG, A0231) from Dako. Anti-mouse and anti-rabbit IgG antibodies conjugated to horseradish peroxidase linked F(ab′)_2_ fragment (from sheep or from donkey respectively) were obtained from Amersham Bioscience. Mouse monoclonal anti-α-tubulin (Clone B-5-1-2), mouse anti-BrdU monoclonal antibody (B2531), and mouse anti-desmosomal protein (ZK-31) were obtained from Sigma Chemical Co. Anti-StarD7Ct was generated in our laboratory as described previously [5]. Two sets of double-stranded siRNA (designated StarD7.1 siRNA and StarD7.2 siRNA) were designed to target the position bases of the StarD7 coding sequence as indicated in Table 1 (Accession No. NM_020151). A scrambled siRNA sequence was used as a negative control.

### Cell Culture and Knockdown of Endogenous StarD7

The human choriocarcinoma cell line JEG-3 (ATCC, HTB-36) was purchased from the American Type Culture Collection (ATCC, Rockville, USA) and cultured in Dulbecco’s modified Eagle’s medium (DMEM), 10% (v/v) fetal bovine serum (FBS), 100 µg/ml penicillin, 100 µg/ml streptomycin (Invitrogen). The cells were harvested and seeded in 6 multiwell plates at 2×10^5^ cells/well. After 24 h and at 40–50% confluency, cells were transfected (Lipofectamine 2000, Invitrogen) with 100 to 200 nM StarD7.1 or StarD7.2 siRNAs (Table 1), to knockdown endogenous StarD7 expression, or scrambled negative siRNA control (Silencer Negative™) (Applied Biosystems/Ambion, Biosystems, Argentina) in 0.5 ml of Opti-MEM serum-free medium. Following 6 h of transfection, 1.5 ml of DMEM was added and cells were cultured for 24–96 h. Culture medium was refreshed every 24 hours.

### Quantitative Reverse Transcription-PCR

Total RNA was extracted from cultured cells using Trizol (Invitrogen), according to the manufacturer’s instructions. Single-stranded cDNAs were synthesized with random primers (Invitrogen) in 20 µl of reaction. Briefly, one µg of total RNA was incubated with these primers (1.25 ng/µl), and the reverse transcriptase reaction was performed as previously described [4].

For real-time PCR, cDNA was mixed with 1× SYBR Green PCR Master Mix (Applied Biosystems) and the forward and reverse primers were added to a final volume of 15 µl. Primer sequences and concentrations used are listed in Table 2. Real-time PCR was carried out on an ABI 7500, Applied Biosystems with Sequence Detection Software v1.4. The cycling conditions included a hot start at 95°C for 10 min, followed by 40 cycles at 95°C for 15 s and 60°C for 1 min. Specificity was verified by melting curve analysis and agarose gel electrophoresis. Each sample was analyzed in triplicate. Transcript levels were normalized to those of cyclophilin A and relative expression levels were calculated using the 2^–ΔΔCt^ method [50]. Amplification efficiency for each set of primers was near 98%. RNA samples incubated without reverse transcriptase during cDNA synthesis, as well as PCR reactions using water instead of template showed no amplification.

**Table 2:** Primer sequences and concentrations used in quantitative RT-PCR.

	Sequence (5′–3′)	nM
**StarD7**		
Sense	GGTAATCAAGCTGGAGGTGATTG	100
Antisense	GAGTACATTGGATAAGGAAAATGGGT	100
**Cyclophilin A**		
Sense	GTCAACCCCACCGTGTTCTT	100
Antisense	CTGCTGTCTTTGGGACCTTGT	100
**βhCG**		
Sense	GCTACTGCCCCACCATGACC	300
Antisense	ATGGACTCGAAGCGCACATC	300
**Syncytin1**		
Sense	GCAACCACGAACGGACATC	150
Antisense	GTATCCAAGACTCCACTCCAGC	150
**ABCG2**		
Sense	CAATGGGATCATGAAACCTG	100
Antisense	CATTTATCAGAACATCTCCAGA	100

### SDS-PAGE and Western Blotting

Protein samples were loaded onto a 10% SDS-PAGE gel. After migration, proteins were electrotransferred to nitrocellulose (Amersham Bioscience). The membrane was blocked in Tris buffered saline (25 mM Tris, 150 mM NaCl, 2 mM KCl, pH 7.4) containing 0.2% Tween 20 and 5% non-fat dry milk, washed and incubated with each one of the following primary antibodies: anti-StarD7Ct (0.5 µg/ml), mouse monoclonal anti-α-tubulin (1∶3000), rabbit polyclonal anti-βhCG (1∶1000), and mouse monoclonal anti-human ABCG2 (1∶300) for 1 h at room temperature with shaking, or as indicated by the manufactures. After washing, the blots were incubated with horseradish peroxidase-conjugated donkey anti-rabbit or sheep anti-mouse secondary antibodies (1∶5000) at room temperature for 1 h. Protein-antibody complexes were visualized by an enhanced chemiluminescence detection system (SuperSignal West Pico; Pierce). Blots were quantified by densitometry using Gel-Pro Analyzer. Protein expression was normalized to the α-tubulin expression.

### βhCG Secretion

JEG-3 cells were treated with scrambled siRNA or StarD7 siRNA and cultured for 72 hours, with the media being replenished once at 24 hours after initial seeding. After 48 or 72 hours, the media was collected and the amount of secreted βhCG was quantified by a solid phase, two-site chemiluminescent immunometric assay with a detection limit of 1.0 mUI/ml (Siemens Immulite 2000).

### Cell Migration Assay

JEG-3 cell migration was measured by determining the ability of the cells to move into an acellular space. The cells were treated with siRNA against StarD7 or scrambled siRNA as mentioned above. After 72 h of culture, the confluent monolayers were wounded using a sterile pipette tip and evaluated under phase contrast microscopy at 0, 8, and 24 h. Photographs were taken and the relative distance traveled by the cells at the acellular front was measured.

Alternatively, cell migration assay was carried out using the Transwell system (Cole Parmer) equipped with 8-mm pore size polycarbonate filters. Cells transfected with scrambled or StarD7 siRNA and cultured during 24 h were trypsinized, resuspended in their basal media containing 10% FBS and loaded onto the upper compartment of Transwell. The chambers were then placed into 24-well culture plates containing the basal media supplemented with 10% FBS. After 48 h of incubation, the non-migrating cells remaining in the upper compartment were removed using cotton swabs and the cells that had migrated to the lower surface of the filters were fixed with methanol and stained with Hoechst 33258. For each experiment, the number of cells in seven randomly chosen fields of each filter was counted. The results of three independent experiments are presented as percentage of cell migration relative to control.

**Cell Proliferation Assay by Bromodeoxyuridine Uptake.**

Proliferation was evaluated by analyzing the incorporation of BrdU into DNA. Cells were treated with siRNA against StarD7 or scrambled siRNA and cultured on coverslips as described above. After 72 h of culture, cells were exposed to 20 µM BrdU (Sigma) for 8 h, washed three times with phosphate-buffered saline (PBS), fixed 10 min in 3% paraformaldehyde, and washed three times with PBS. Cells were permeabilized for 10 min with 0.01% Triton X-100 in PBS (PBSTx). Cells were washed with PBS containing Tween 0.2% (PBST), treated with 2 N HCl at 37°C for 30 min, washed two times with 0.1 M NaB_4_O_7_, and then three times with PBSTx. Cells were resuspended in PBS containing 0.5% Tween 20 and 1% bovine serum albumin and incubated with mouse anti-BrdU monoclonal antibody at 1∶100 dilution for 1 h at 37°C. Then, cells were washed three times with PBST and incubated 1 h with Alexa Fluor 594-conjugated goat anti-mouse IgG (1∶1000). Cells were washed with PBST and slides were mounted in Aqueous Mounting Medium with fluorescence 361 tracers (Fluor Safe, Calbiochem).

### Cell Proliferation by [^3^H]-thymidine Incorporation Assay

To measure the effect of StarD7 siRNA on cell proliferation, cells transfected either with scrambled or StarD7 siRNA were plated in quintuplicate at 1.5×10^4^ cells/well in a 96-well plate, cultured during 72 h, and pulsed with [5′-^3^H] thymidine (20 µCi/ml, specific activity: 20 Ci/mmol) for the last 18 h of culture. Cells were harvested and washed with PBS. DNA was precipitated with cold 10% trichloroacetic acid (TCA), redissolved in 0.1 M NaOH with 1% SDS at 37°C for 1 h, and the radioactivity of the samples was counted in a Beckman liquid scintillation counter.

### Immunofluorescence and Cell-fusion Assay

JEG-3 cells treated with siRNA against StarD7 or scrambled siRNA were cultured on coverslips as described above. Cells were fixed 10 min in cold methanol and incubated 10 min with 1 mM ammonium chloride to inhibit quenching. Cells were permeabilized for 7 min with PBSTx. Cells were then rinsed PBS three times, blocked with 2.5% normal goat serum in PBST and with 0.2% fish skin gelatin in PBSTx, and then incubated at 37°C with the mouse anti-desmosomal protein (1∶400) or mouse monoclonal anti-human ABCG2 (1∶100). Cells were washed with PBSTx and incubated 1 h with Alexa Fluor 594-conjugated goat anti-mouse IgG and nuclei were counterstained with Hoechst 33258 dye. Cells were washed with PBST and slides were mounted in Aqueous Mounting Medium with fluorescence 361 tracers (Fluor Safe, Calbiochem). Intercellular fusion was quantified by observing the coverslips using fluorescent microscopy, merging the Alexa Fluor 594 and Hoechst images, and counting the number of nuclei in syncytia and the total number of nuclei in twenty randomly chosen microscopic fields. The percentage of the nuclei in syncytia was determined as: (number of nuclei in syncytia/total number of nuclei) ×100, as described [51]. Duplicate wells were evaluated in each experiment, and each experiment was performed at least three times independently. Observations of cells were made in an inverted microscope Nikon Eclipse TE 2000U (Nikon Corporation, Japan) or a Confocal Olympus FLuoview FV300 microscope (Olympus Latin America, Miami, FL), as indicated.

### Phospholipid Synthesis

The incorporation of ^3^H-glycerol into phospholipids in JEG-3 cells transfected with StarD7 siRNA or scrambled siRNA was assessed after 48 h of transfection. A 3 h labelling pulse of [2-^3^H]-glycerol (2 µCi/well; specific activity: 200 mCi/mmol) was given to cultures of each condition. The radioactive phospholipids were determined as described [52], [53]. In brief, cell preparations washed twice with 1 mM cold PBS were resuspended in 1 ml of water and precipitated with the same volume of 10% TCA and 1% phosphotungstic acid. After centrifugation (800×g, 15 min), the supernatant fraction was separated. Pellets were washed three times with 5% TCA and 0.5% phosphotungstic acid and once with water by successive resuspension and centrifugation. Phospholipids were extracted with chloroform/methanol (2∶1 vol/vol), and radioactivity was determined in a liquid scintillation counter. The content of protein in cell preparations was estimated by Bradford [54], using bovine serum albumin as standard. Individual phospholipids were separated by a 1-D two-solvent system procedure using high performance-TLC silica gel 60-precoated sheets with UV detector (Macherey-Nagel; Duren, Germany) as described by Weiss et al. [55]. Standards and labelled individual lipid species were visualized with iodine vapors, scraped from the silica-plate and the radioactivity in each lipid was quantified in a liquid scintillation counter.

### Data Analysis

Significant differences for control and test conditions were identified using the nonparametric paired Wilcoxon test or unpaired Mann-Whitney U-test test. A Kruskal–Wallis with a Dunns post-test was performed to obtain a multiple comparison of independent sample populations. Significance was taken as *p<*0.05.
